# Regulation of Oxidative Stress by Long Non-Coding RNAs in Vascular Complications of Diabetes

**DOI:** 10.3390/life12020274

**Published:** 2022-02-12

**Authors:** Pei-Ming Chu, Cheng-Chia Yu, Kun-Ling Tsai, Pei-Ling Hsieh

**Affiliations:** 1 Department of Anatomy, School of Medicine, China Medical University, Taichung 404333, Taiwan; pmchu@mail.cmu.edu.tw; 2 Institute of Oral Sciences, Chung Shan Medical University, Taichung 40201, Taiwan; ccyu@csmu.edu.tw; 3 Department of Dentistry, Chung Shan Medical University Hospital, Taichung 40201, Taiwan; 4 School of Dentistry, Chung Shan Medical University, Taichung 40201, Taiwan; 5 Department of Physical Therapy, College of Medicine, National Cheng Kung University, Tainan 70101, Taiwan; kunlingtsai@mail.ncku.edu.tw; 6 Institute of Allied Health Sciences, College of Medicine, National Cheng Kung University, Tainan 70101, Taiwan

**Keywords:** diabetic vascular complication, oxidative stress, long non-coding RNA

## Abstract

Diabetes mellitus is a well-known metabolic disorder with numerous complications, such as macrovascular diseases (e.g., coronary heart disease, diabetic cardiomyopathy, stroke, and peripheral vascular disease), microvascular diseases (e.g., diabetic nephropathy, retinopathy, and diabetic cataract), and neuropathy. Multiple contributing factors are implicated in these complications, and the accumulation of oxidative stress is one of the critical ones. Several lines of evidence have suggested that oxidative stress may induce epigenetic modifications that eventually contribute to diabetic vascular complications. As one kind of epigenetic regulator involved in various disorders, non-coding RNAs have received great attention over the past few years. Non-coding RNAs can be roughly divided into short (such as microRNAs; ~21–25 nucleotides) or long non-coding RNAs (lncRNAs; >200 nucleotides). In this review, we briefly discussed the research regarding the roles of various lncRNAs, such as MALAT1, MEG3, GAS5, SNHG16, CASC2, HOTAIR, in the development of diabetic vascular complications in response to the stimulation of oxidative stress.

## 1. Diabetes Mellitus and Oxidative Stress

Diabetes mellitus (DM) is a chronic metabolic disorder with an increasing risk of cardiovascular diseases. It has been revealed that the global prevalence of DM is approximately 9.3% (463 million people) in 2019 and estimated to be 10.2% (578 million) by 2030 and 10.9% (700 million) by 2045 [1]. There are several types of DM, including type I (insulin-dependent, juvenile DM), type II (non-insulin-dependent, adult-onset DM), and gestational DM [2]. DM has a well-established association with an increase in the likelihood of developing various complications, such as macrovascular and microvascular diseases. Macrovascular disorders include coronary heart disease, diabetic cardiomyopathy, stroke, and peripheral vascular disease, and microvascular disorders comprise diabetic nephropathy and retinopathy. Although the mortality rates owing to vascular diseases have declined, it remains one of the leading causes of death in subjects with diabetes [3,4]. Due to the rising prevalence and diverse complications, DM represents a substantial health challenge that affects the quality of life of patients and elevates the demands on health services.

Oxidative stress refers to the enhanced intracellular levels of reactive oxygen species (ROS), such as superoxide anion (O_2_^−^), hydrogen peroxide (H_2_O_2_), and hydroxyl radicals (OH^·^), that are not fully eliminated by certain antioxidant enzymes, such as superoxide dismutases (SOD), and then induce damage to lipids, proteins, and DNA. To regulate the cellular redox balance and protective antioxidant system, the nuclear factor erythroid 2-related factor 2 (Nrf2) and its target genes are activated. Under physiological conditions, Kelch-like ECH-associated protein 1 (Keap1) is responsible for the cytosolic sequestration of Nrf2. Under stressful conditions, Nrf2 will be released from Keap1 and translocate into the nucleus where it binds to the antioxidant response element (ARE) sequence and increases the transcription of antioxidant enzymes, such as heme oxygenase-1 (HO-1) [5]. On the other hand, the hyperglycemia-induced tissue injury has been shown to be mediated by the following mechanisms, including the increased flux of glucose and other sugars through the polyol pathway, the increased production of advanced glycation end products (AGEs), activation of protein kinase C (PKC) isoforms, increased hexosamine pathway flux and consequent over-modification of proteins by N-acetylglucosamine, and these pathways are all activated by the hyperglycemia-induced mitochondrial superoxide production by inhibiting GAPDH [6]. Accordingly, the downregulation of hyperglycemia-associated oxidative stress will aid in the better control of diabetic complications. 

## 2. Long Non-Coding RNA

Emerging evidence has suggested that non-coding RNAs may function as crucial modulators in the response to oxidative stress [7]. Non-coding RNAs constitute the vast majority of human transcripts and are classified into different groups based on their length and function. It usually can be categorized into short (such as microRNAs; ~21–25 nucleotides) or long non-coding RNAs (lncRNAs; >200 nucleotides). Unlike microRNAs which exert their regulatory capacity through binding to the 3′ untranslated region (UTR) of their target genes, the embodiment of the lncRNAs function includes a variety of ways, such as serving as scaffolds, molecular signals, guides, or decoys [8]. Recently, more and more lncRNAs have been discovered to shape gene expression by titrating microRNAs with their microRNAs response elements (MREs), and they are called competing endogenous RNAs (ceRNAs) [9]. These ceRNAs act as natural microRNAs sponges and may bind multiple microRNA molecules to co-regulate each other.

**Figure 1:**
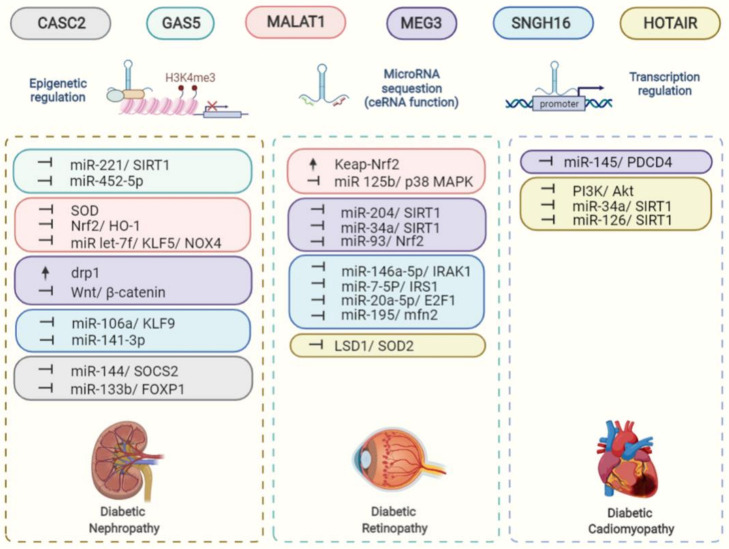
Roles and functions of the listed lncRNAs in the regulation of oxidative stress-related complications.

Aberrant expression of lncRNAs has been observed in various diseases, including in samples of DM patients [10]. Besides, lncRNAs are involved in the development of insulin resistance by regulating lipid and carbohydrate metabolism, inflammatory process, and oxidative stress [11]. Of note, a variety of lncRNAs have functional interactions with microRNAs by serving as molecular sponges to affect several aspects of cellular responses to oxidative stress. In this review, we discuss the roles of multiple lncRNAs in a number of diabetic complications and how they modulate oxidative stress insult. We aim to provide better insight into the association between the dysregulation of lncRNAs and the accumulation of oxidative stress in disease progression. In the following sections, we summarize various lncRNAs (e.g., HOTAIR, MALAT1, MEG3, GAS5, SNHG16, and CASC2) that contribute to the development of these diabetic vascular complications through regulation of oxidative stress (Figure 1).

## 3. Diabetic Nephropathy

The deterioration of kidney function due to DM is called diabetic nephropathy, which has been known as the most common cause of end-stage renal disease (ESRD) [12]. The cellular components of the kidney, such as glomerular endothelial cells, mesangial cells, podocytes, and tubular epithelial cells, are potential targets of hyperglycemic injury. Patients with diabetic nephropathy may experience glomerular hyperfiltration, progressive albuminuria, reduction in glomerular filtration rate, the elevation of arterial blood pressure and fluid retention, and ultimately, ESRD. Numerous pathways have been revealed to be activated during the course of diabetic nephropathy, such as the renin-angiotensin-aldosterone system; the pro-fibrotic and inflammatory cytokines, such as transforming growth factor β (TGF-β) and tumor necrosis factor-α (TNF-α); various kinases such as PKC; and oxidative stress mediators, such as nicotinamide adenine dinucleotide phosphate oxidase (NADPH oxidase) [13]. 

### 3.1. HOTAIR

HOX antisense intergenic RNA (HOTAIR) is a 2.2 kilobase non-coding RNA and one of the first described lncRNA [14]. It is located in the homeobox C gene (HOXC) cluster between the *HOXC11* and *HOXC12* genes on human chromosome 12q13.13. HOTAIR is an example of scaffold lncRNAs as it interacts with Polycomb Repressive Complex 2 (PRC2), which methylates histone H3 on lysine 27 to induce gene repression [14]. HOTAIR also exerts its transcriptional effect across chromosomes in trans, and has been found to promote breast cancer metastasis [15]. HOTAIR is expressed in normal adult human kidney tissue, including glomerular podocytes. It also has been found to be upregulated in the kidneys of humans and mice with diabetes [16]. Nevertheless, Majumder et al. showed that knockout of HOTAIR from podocytes has minimal effect on the glomerular injury in diabetic mice [16]. They concluded that the dysregulation of HOTAIR in diabetes may be a bystander in diabetic kidney disease instead of a contributor. 

### 3.2. MALAT1

Metastasis-associated lung adenocarcinoma transcript 1 (MALAT1), also known as NEAT2, is an 8.5 kilobase lncRNA located within the human chromosome 11q13 [17]. As its name suggests, MALAT1 serves as an oncogene in various types of cancers, and it has been found to be increased in kidney cortices from C57BL/6 mice with streptozocin (STZ)-induced type I DM [18]. In the work by Hu et al., they showed that there is a feedback regulation between MALAT1 and β-catenin, which may implicate in the high glucose-induced podocyte damage [18]. Likewise, the circulating expression of MALAT1 is upregulated in patients with diabetic nephropathy, and there is a negative correlation between MALAT1 and the anti-oxidant enzyme, SOD [19]. It has been shown that the silencing of MALAT1 downregulates the oxidative stress and inflammatory reaction in the renal tissues of diabetes (db/db) mice [20]. Moreover, MALAT1 has been demonstrated to contribute to the podocyte injury by reducing miR let-7f and increasing *krüppel-like factor 5* (KLF5) [20]. KLF5 has been found to induce oxidative stress via directly binding on NADPH oxidase (NOX)4 promoter, which elevates the expression of NOX4 in AC16 human cardiomyocyte cells [21]. Hence, MALAT1 may also generate oxidative stress in diabetic renal tissues by regulating KLF5/ NOX4 signaling. Additionally, it has been demonstrated that the expression levels of Nrf2 and HO-1 are both elevated in mouse podocyte MPC-5 cells with MALAT1-3 siRNA under high glucose conditions [22]. As mentioned above, Nrf2 is a redox-sensitive transcription factor, which regulates the expression of various antioxidant enzymes via ARE [23]. As such, another avenue for MALAT1 to generate oxidative stress is via modulation of Nrf2/HO-1 signaling.

### 3.3. MEG3

Maternally expressed gene 3 (MEG3), also known as gene trap locus 2 (Gtl2), is located at chromosome 14q32.3 within *Dlk1-Dio3* locus [24,25]. Knockdown of MEG3 has been found to potentiate insulin resistance and impair glucose homeostasis in diet-induced obese mice [26]. It has been shown that the expression of MEG3 is increased in podocytes of the STZ-induced mice, high glucose-stimulated human podocytes and mesangial cells, and serum of patients with diabetic nephropathy [27,28,29]. Moreover, knockdown of MEG3 in podocytes alleviates albuminuria, renal dysfunction, glomerular injury, and podocyte mitochondrial fission in DM mice [27]. The in vitro experiment shows that MEG3 promotes mitochondrial fission in podocytes by regulation of dynamin-1-like protein (drp1) and its translocation to mitochondria [27], which may cause oxidative stress and cell damage in DM [30,31]. MEG3 has been proven to interact with several microRNAs, such as miR-181a [28] and miR-145 [29], that are associated with the regulation of oxidative stress [32,33]. However, one study with controversial findings suggests that MEG3 expression is decreased in renal tissues of patients with DN and podocytes treated with high glucose [34]. They demonstrated that MEG3 protects against the high glucose-induced ROS production and podocyte injury via inactivating Wnt/β-catenin signaling [34].

### 3.4. GAS5

Growth arrest-specific 5 (GAS5) is located at 1q25.1 and was originally identified in a study aimed to screen for novel tumor suppressor genes expressed at high levels during growth arrest [35]. It has been shown that GAS5 expression is reduced in high glucose-induced proximal tubular cells [36] or mesangial cells [37]. In the kidney tissues of type 2 DM with diabetic nephropathy, GAS5 is also downregulated compared with that in patients without diabetic nephropathy [37]. Nevertheless, the animal studies seem to exhibit inconsistent results. It has been shown that GAS5 is increased in kidneys of the high-fat diet (HFD)/STZ-induced diabetic mice and reported to exacerbate renal tubular epithelial fibrosis [38]. However, other studies demonstrate that the expression of GAS5 in kidney tissues of STZ-induced diabetic nephropathy rats is decreased, which may alleviate renal fibrosis in diabetic nephropathy [37,39]. In addition to suppression of matrix metalloproteinase 9 via recruiting enhancer of zeste homolog 2 (EZH2) [39], GAS5 also improves the high glucose-induced renal damage by reduction of oxidative stress. 

It has been shown that GAS5 can serve as a ceRNA to sponge miR-221 via both directly targeting and Ago2-dependent manner. The work by Ge et al. demonstrates that miR-221 is able to downregulate the proliferation and fibrosis-related proteins by targeting sirtuin1 (SIRT1) [37]. Given that SIRT1 is a protein deacetylase that helps to antagonize oxidative stress in various disease conditions, including diabetes [40,41], it is reasonable to postulate that upregulation of GAS5 may downregulate the increased oxidative stress in diabetic nephropathy. This assumption proves to be correct as another study demonstrates that GAS5 mitigates oxidative stress in high-glucose-stimulated renal tubular cells [36]. They show overexpression of GAS5 reverses the increased ROS and oxidative stress indicator, malondialdehyde (MDA) as well as the anti-oxidant SOD in high-glucose-stimulated cells via directly binding to miR-452-5p [36].

### 3.5. SNHG16

The small nucleolar RNA host genes (SNHGs) are a group of lncRNAs, including SNHG1, SNHG3, SNHG5, SNHG6, SNHG7, SNHG12, SNHG15, SNHG16, SNHG20, that are found to exhibit oncogenic roles in a variety of malignancies [42]. SNHG16, also known as non-coding RNA, expressed in aggressive neuroblastoma (ncRAN), is located at chromosome 17q25.1 [43]. It has been revealed that SNHG16 is overexpressed in the serum of diabetic nephropathy patients, high glucose-treated podocytes [44], and mesangial cells [45]. In the renal cortex of db/db mice, SNHG16 is markedly increased as well [45]. It has been shown that SNHG16 is involved in the proliferation of mesangial cells and fibrogenesis via modulating miR-141-3p [45]. Besides, He et al. show that SNHG16 aggravates the high glucose-induced podocytes injury, including cell viability, ROS production, and apoptosis, through upregulation of *Krüppel-like factor 9* (KLF9) via sponging miR-106a [44]. KLF9 has been known to bind to the promoters and affects the expression of various genes involved in the metabolism of ROS, such as the anti-oxidant defense gene thioredoxin reductase 2, and Nrf2 can exaggerate oxidative injury through induction of KLF9 [46]. Therefore, SNHG16 seems to contribute to diabetic nephropathy via KLF9-induced oxidative stress by directly interfering with miR-106a.

### 3.6. CASC2

Cancer susceptibility candidate 2 (CASC2) is located at chromosome 10q26 in humans and was first discovered in 2004 by Baldinu et al. [47]. CASC2 acts as a tumor suppressor in various types of cancers [48] and its expression level is reduced in the serum of diabetic nephropathy patients [49], tissues in db/db diabetic mouse, and high glucose-treated human renal mesangial cells [50] and podocyte cells [49,50,51,52]. Upregulation of CASC2 has been shown to attenuate the progression of diabetic nephropathy via various pathways. For instance, it has been shown that overexpression of CASC2 inhibits apoptosis of podocyte cells and reduces the phosphorylation level of c-Jun N-terminal kinase 1 (JNK1) [49]. CASC2 also mitigates cell apoptosis, inflammatory factor release, and fibrosis progression via miR-144/suppressor of cytokine signaling 2 (SOCS2) axis [50]. Although the role of SOCS2 in the regulation of oxidative stress in diabetic nephropathy has not been fully elucidated, SOCS2 has been revealed to modulate ROS formation in hepatocytes [53]. Besides, upregulation of CASC2 downregulates the high glucose-induced proliferation, extracellular matrix deposition and oxidative stress of human mesangial cells via miR-133b/ forkhead box P1 (FOXP1) axis [52]. It has been demonstrated that overexpression of FOXP1 reduces ROS level through downregulation of Akt/mTOR signaling and the expression of two major ROS-producing enzymes, NOX2 and NOX4 [54].

## 4. Diabetic Cardiomyopathy and Vascular Complications

Diabetic cardiomyopathy refers to the presence of abnormal myocardial structure or performance in the absence of other risk factors, such as coronary artery disease, hypertension, and significant valvular disease, in individuals with DM [55]. It is characterized by left ventricular hypertrophy, dysfunctional remodeling, diastolic and systolic dysfunction, and eventually heart failure [56]. Among various mechanisms that are implicated in the development of diabetic cardiomyopathy, it has been demonstrated that H19/miR-675 axis modulates apoptosis of cardiomyocytes by affecting voltage-dependent anion channel 1 (VDAC1) [57]. Moreover, the increased ROS has been shown to impair cardiac structure by directly damaging myocytes. For example, it has been demonstrated that short-term exposure to H_2_O_2_ of rat ventricular myocytes results in a progressive decrease in cell shortening followed by diastolic arrest, which may be due to oxidative modification of sarcoendoplasmic reticulum Ca^2+^-ATPase (SERCA) and the Na^+^/Ca^2+^ exchanger (NCX) [58]. Besides, increased cardiomyocyte NOX2 activity has been observed in diet-induced obese female mice with cardiac diastolic dysfunction [59].

### 4.1. HOTAIR

Although HOTAIR has been considered as an oncogene in various types of cancers [60], it seems to possess a protective effect in patients with diabetic cardiomyopathy. It has been reported that HOTAIR expression is reduced in myocardial tissues and serum of diabetic cardiomyopathy patients [61]. Moreover, HOTAIR has been proven to increase the viability of cardiomyocytes, possibly through activation of the PI3K/Akt pathway [61]. Various studies have shown that the PI3K-Akt pathway is associated with oxidative stress generation in numerous diabetic impairments [62,63]. Similar findings are presented in the STZ-induced type 1 DM model. In diabetic mice hearts, the HOTAIR expression is significantly decreased. Moreover, knockdown of HOTAIR in the high glucose-treated cardiomyocytes H9c2 cells leads to increased oxidative injury, inflammation, and apoptosis. Gao et al. show that HOTAIR acts as a molecular sponge of miR-34a, which subsequently activates the expression of SIRT1 by inhibiting the repression from miR-34a [64]. Overexpression of HOTAIR also has been demonstrated to inhibit the expression of miR-126, which increases the expression of SIRT1 by regulating Klotho in aortic smooth muscle cells [65].

### 4.2. MEG3

MEG3 has been found to be overexpressed in high glucose-treated cardiomyocytes and serve as a ceRNA for miR-145 to induce apoptosis by upregulating the expression of programmed cell death 4 (PDCD4) [66]. Given that miR-145 has been suggested to mitigate the high glucose-induced oxidative stress and inflammation in retinal endothelial cells [67], it is tempting to assume that MEG3 may enhance the accumulation of oxidative stress via directly binding to miR-145. Of note, MEG3 deficiency has been shown to cause impairment of insulin signaling through induction of cellular senescence in the hepatic endothelium. It has been revealed that the expression of endothelial MEG3 is elevated in obesity, and knockdown of MEG3 causes cellular senescence in human umbilical vein endothelial cells (HUVECs) and hepatic endothelium in obese mice [26]. In addition, MEG3 knockdown upregulates the level of superoxide and other free radicals in HUVECs using MitoSOX staining and electron paramagnetic resonance spectroscopy. The work by Cheng et al. demonstrated that lacking MEG3 decreases the basal mitochondrial O_2_ consumption rate and the maximal respiration capacity without affecting the extracellular acidification rate [26].

## 5. Diabetic Retinopathy and Diabetic Cataract

Diabetic retinopathy is one of the most common causes of blindness among adults and the elderly. Nearly all patients with type I DM and more than 60% of patients with type II DM eventually have diabetic retinopathy [68]. There are two kinds of diabetic retinopathy, non-proliferative and proliferative diabetic retinopathies. Non-proliferative diabetic retinopathy is characterized by vascular closure, preceded by increased vascular permeability. As the disease progresses into proliferative diabetic retinopathy, abnormal growth of blood vessels on the retina in response to the ischemia may cause blood leak into the vitreous which leads to the subsequent macular edema [68]. It has been shown that lncRNA H19 inhibits the endothelial-mesenchymal transition of retinal endothelial cells [69] and regulates inflammation of retinal epithelial cells [70] in diabetic retinopathy. Luo et al. showed that H19 can sponge miR-93 and modulate X-box binding protein 1 (XBP1) to downregulate pro-inflammatory cytokines [70]. On the other hand, several studies have demonstrated that supplements of anti-oxidant enzymes could attenuate the progression of the diabetic retina [71] and inhibit the activation of redox-sensitive nuclear transcriptional factors [72]. In the following sections, we summarized how lncRNAs are involved in the regulation of abnormal retinal metabolism in diabetes.

### 5.1. HOTAIR

It is reported that there is a significant increase in serum HOTAIR of diabetic retinopathy patients, and the upregulation of HOTAIR can be used to discriminate diabetic retinopathy from nondiabetic retinopathy [73]. One of the in vitro studies supports this finding and shows that high glucose markedly augments the expression of HOTAIR in human retinal endothelial cells, which increases oxidative damage, endothelial cell junction disruptions, and mitochondrial aberrations [74]. Another study demonstrated that HOTAIR can directly interact with lysine-specific histone demethylase 1 (LSD1) using retinal endothelial cells [75]. It has been shown that high glucose enhances the binding of LSD1 and Sp1 at SOD2 (MnSOD), and knockdown of LSD1 with siRNA improves the high glucose-induced H3K4 demethylation at SOD2 to increase *SOD2* gene expression [76]. These findings suggest that HOTAIR may regulate the antioxidant capacity in the development of diabetic retinopathy by affecting the epigenetic modification of SOD2.

### 5.2. MALAT1

The aberrant upregulation of MALAT1 has been reported in the diabetic retinas using clinical samples, STZ-induced type I DM mice [77], and db/db type 2 DM mice [78]. In the MALAT1 null mice, the expression of Nrf2 and antioxidant genes including Nqo1 and Cat is upregulated with concomitant downregulation of ROS and ROS-generated protein carbonylation in hepatocyte and islets [79]. Chen et al. demonstrated that MALAT1 interacts with Nrf2 and regulates insulin resistance by modulation of JNK activity and phosphorylation of Akt [79]. In diabetic retinopathy, MALAT1 also plays an integral part in the regulation of the Keap1-Nrf2-antioxidant defense system. It has been revealed that high glucose elevates the expression of MALAT1 by increasing Sp1 transcription factor binding at the MALAT1 promoter. Furthermore, the increased MALAT1 recruits more Sp1 at the Keap1 promoter and activates its transcription [80]. Given that Keap1 quenches Nrf2 and inhibits its transcriptional activity, the transcription of the antioxidant response enzymes, such as HO1 and SOD2, are also decreased [80].

Another study showed that MALAT1 competitively binds to miR-125b against vascular endothelial-cadherin, which may promote neovascularization in diabetic retinopathy [81]. Although there is a lack of direct evidence showing MALAT1/ miR-125b axis affects oxidative stress, it is reported that ROS production in neuronal PC12 cells is remarkably upregulated upon transfection with the miR-125b-5p mimic under high-glucose conditions [82]. Whether the interaction between MALAT1 and miR-125b contributes to the redox homeostasis in diabetic retinopathy is worthy of investigation.

Besides, it has been shown that knockdown of MALAT1 ameliorates the retinal function and retinal vessel impairment in diabetic rats. Moreover, the cell viability, migration and tube formation, retinal inflammation, oxidative stress in retinal endothelial cells are improved. The work by Liu et al. suggests that MALAT1 knockdown prevents the hyper-proliferation of retinal endothelial cells through p38 mitogen-activated protein kinase (MAPK) signaling [78]. A similar finding is reported in diabetic cataracts. It is revealed that MALAT1 is aberrantly expressed in the anterior lens capsule tissues of patients with diabetic cataracts and high glucose-treated human lens epithelial cells [83]. Moreover, knockdown of MALAT1 also reverses the high glucose-induced oxidative stress, including a higher level of oxidative stress indicator MDA and lower levels of anti-oxidant SOD and glutathione peroxidase (GSH-PX) via the p38MAPK pathway [83]. Taken together, MALAT1 seems to modulate ROS via regulation of the Keap1-Nrf2 axis and p38MAPK pathway.

### 5.3. MEG3

It has been shown that MEG3 expression level is markedly downregulated in the fibrovascular membranes of diabetic patients and retinas of STZ-induced diabetic mice [84] as well as the high glucose-treated Müller cells, the principal glial cell of the retina [85]. MEG3 has been shown to act as a ceRNA for miR-204 and counteract its suppression of SIRT1 [85]. The subsequent work by Tu et al. demonstrated that deacetylation of SIRT1 target genes, such as forkhead box o1 (FOXO1) and nuclear factor kappa B (NF-κB) subunit p65, eventually leads to the reduction of oxidative stress and inflammation in high glucose-treated Müller cells [86]. In human retinal pigment epithelium cells, MEG3 has been demonstrated to increase Nrf2 by sequestering miR-93, which suppresses high glucose-induced apoptosis and inflammation [87]. Similarly, MEG3 can alleviate high glucose-associated apoptosis and inflammation through inhibiting NF-κB signaling via impeding the interaction between miR-34a and SIRT1 in retina epithelial cells [88]. These results show that MEG3 may be beneficial to treat diabetic retinopathy by suppression of oxidative stress and inflammation.

### 5.4. SNHG16

Several studies indicate that SNHG16 aggravates diabetic retinopathy. It has been demonstrated that SNHG16 is markedly upregulated in H_2_O_2_, high glucose, or AGE-treated human retinal microvascular endothelial cells (hRMECs) [89,90]. Cai et al. show that an increase of SNHG16 in the high glucose-stimulated hRMECs promotes the proliferative diabetic retinopathy-related abnormalities in cell proliferation, migration, and angiogenesis via modulation of miR-146a-5p/interleukin-1 receptor-associated kinase 1 (IRAK1) and miR-7-5p/insulin receptor substrate 1 (IRS1) to activate NF-κB and PI3K/AKT signaling pathways, respectively [89]. Another study reveals that SNHG16 modulates the expression of E2F transcription factor-1 (E2F1) by sponging miR-20a-5p, which increases apoptosis of hRMECs [91]. E2F1 has been shown to regulate oxidative metabolism [92] and mediate the direct effects of high glucose on retinal neurons, glial cells, and blood vessels [93]. Besides, SNHG16 also increases oxidative stress-induced pathological angiogenesis in hMRECs through modulating the miR-195/ mitofusin 2 (mfn2) axis [90]. Mfn2 implicates in the control of mitochondrial membrane fusion and is downregulated in the diabetic retina [94]. It has been revealed that overexpression of mfn2 attenuates the glucose-induced accumulation of mitochondrial ROS [94]. As such, these results suggest that the high glucose-inhibited SNHG16 may contribute to the increased oxidative stress in DR via miR-20a-5p/E2F1 or miR-195/mfn2 axes. The aforementioned lncRNAs and the associated mechanisms are listed in Table 1.

## 6. Conclusions

DM is a multifactorial disorder with a wide range of vascular complications. As shown in Table 1, the aberrant expression of lncRNAs may act as potential biomarkers for the diagnosis and prognosis of macrovascular and microvascular diseases. We showed that these lncRNAs can directly modulate the anti-oxidant pathways (such as Nrf2/HO-1 signaling) and enzymes (such as SOD). Besides, cumulative research suggests that numerous lncRNAs can regulate the pathogenesis of diabetic vascular complications through interacting with several microRNAs. Further research is needed to investigate if targeting these lncNRAs can effectively alleviate the progression of diabetic vascular complications.

## Figures and Tables

**Table 1:** Summary of the expression and associated mechanisms of lncRNAs in various diabetic complications.

Diabetic Complications	LncRNA	Expression Level	Cells or Tissues	Interacting Factors/Pathways	Ref.
Diabetic Nephropathy	MALAT1	Upregulated	Kidney cortices in STZ-injected C57BL/6 mice		[18]
Diabetic Nephropathy	MALAT1		Patients with DN	SOD	[19]
Diabetic Nephropathy	MALAT1		Mouse podocyte MPC-5 cells	KLF5	[20]
Diabetic Nephropathy	MALAT1		Mouse podocyte MPC-5 cells	Nrf2/HO-1 signaling	[22]
Diabetic Nephropathy	MEG3	Upregulated	Podocytes of STZ-induced mice	drp1	[27]
Diabetic Nephropathy	MEG3	Upregulated	HG-treated mesangial cells	miR-181a	[28]
Diabetic Nephropathy	MEG3	Upregulated	Serum of patients with DN	miR-145	[29]
Diabetic Nephropathy	MEG3	Downregulated	Renal tissues of patients with DN and HG-treated podocytes	Wnt/β-catenin signaling	[34]
Diabetic Nephropathy	GAS5	Downregulated	HG-induced proximal tubular cells	miR-452-5p/SOD axis	[36]
Diabetic Nephropathy	GAS5	Downregulated	HG-treated mesangial cells	miR-221/SIRT1 axis	[37]
Diabetic Nephropathy	GAS5	Upregulated	Kidneys of the HFD/ STZ-induced diabetic mice		[38]
Diabetic Nephropathy	SNHG16	Upregulated	Serum of DN patients and HG-treated podocytes	miR-106a/KLF9 axis	[44]
Diabetic Nephropathy	SNHG16	Upregulated	HG-treated mesangial cells		[45]
Diabetic Nephropathy	CASC2	Downregulated	Serum of DN patients and podocyte cells		[49]
Diabetic Nephropathy	CASC2	Downregulated	Tissues in db/db diabetic mouse and HG-treated mesangial cells and podocytes		[50]
Diabetic Nephropathy	CASC2	Downregulated	HG-treated podocyte cells	miR-133b/FOXP1 axis	[52]
Diabetic Cardiomyopathy	HOTAIR	Downregulated	Myocardial tissues and serum of diabetic cardiomyopathy patients		[61]
Diabetic Cardiomyopathy	HOTAIR	Downregulated	Diabetic hearts in STZ-injected C57/B6 mice andHG-stimulated H9c2 cells	miR-34a/SIRT1 axis	[64]
Diabetic Cardiomyopathy	MEG3	Upregulated	HG-treated cardiomyocytes	miR-145	[66]
Diabetic Retinopathy	HOTAIR	Upregulated	HG-treated human retinal endothelial cells		[74]
Diabetic Retinopathy	HOTAIR		HG-treated human retinal endothelial cells	LSD1/MnSOD axis	[75,76]
Diabetic Retinopathy	MALAT1	Upregulated	Diabetic retinas using clinical samples, STZ-induced type I DM mice		[77]
Diabetic Retinopathy	MALAT1		db/db type 2 DM mice		[78]
Diabetic Retinopathy	MALAT1			Keap1-Nrf2 axis	[79,80]
Diabetic Retinopathy	MALAT1		HG-treated human lens epithelial cells	SP1 and p38MAPK pathway	[83]
Diabetic Retinopathy	MEG3	Downregulated	HG-treated Müller cells	miR-204/SIRT1 axis	[85]
Diabetic Retinopathy	MEG3		HG-treated retinal pigment epithelium cells	miR-93/Nrf2 axis	[87]
Diabetic Retinopathy	MEG3		HG-treated retina epithelial cells	miR-34a/SIRT1 axis	[88]
Diabetic Retinopathy	SNHG16	Upregulated	HG-treated retinal microvascular endothelial cells		[89]
Diabetic Retinopathy	SNHG16		HG-treated retinal microvascular endothelial cells	miR-195/mfn2 axis	[90]
Diabetic Retinopathy	SNHG16		HG-treated retinal microvascular endothelial cells	miR-20a-5p/E2F1 axis	[91]

Abbreviation: STZ, streptozotocin; DN, diabetic nephropathy; SOD, superoxidase dismutase; KLF5/9, Kruppel-like factor 5/9; Nrf2, nuclear factor erythroid 2-related factor 2; HO-1, heme oxygenase-1; Drp1, dynamin-related protein 1; HG, high glucose; SIRT1, Sirtuin 1; HFD, high-fat diet; FOXP1, forkhead box protein 1; LSD1, lysine-specific demethylase 1; keap1, Kelch-like ECH-associated protein 1; SP1, specificity protein 1; Mfn2, mitofusin-2; E2F1, E2F transcription factor 1.
